# Transplant Trial Watch

**DOI:** 10.3389/ti.2024.12597

**Published:** 2024-01-22

**Authors:** John M. O’Callaghan, Simon R. Knight

**Affiliations:** ^1^ University Hospitals Coventry & Warwickshire, Coventry, United Kingdom; ^2^ Centre for Evidence in Transplantation, Nuffield Department of Surgical Sciences, University of Oxford, Oxford, United Kingdom; ^3^ Oxford Transplant Centre, Churchill Hospital, Oxford, United Kingdom

**Keywords:** randomised controlled trial, kideny transplantation, solid organ transplant (SOT), tacrolimus, cytomegalovirus

To keep the transplantation community informed about recently published level 1 evidence in organ transplantation ESOT and the Centre for Evidence in Transplantation have developed the Transplant Trial Watch. The Transplant Trial Watch is a monthly overview of 10 new randomised controlled trials (RCTs) and systematic reviews. This page of Transplant International offers commentaries on methodological issues and clinical implications on two articles of particular interest from the CET Transplant Trial Watch monthly selection. For all high quality evidence in solid organ transplantation, visit the Transplant Library: www.transplantlibrary.com.

RANDOMISED CONTROLLED TRIAL 1 A prospective controlled, randomized clinical trial of kidney transplant recipients developed personalized tacrolimus dosing using model-based Bayesian Prediction.

*by Lloberas, N., et al. Kidney International 2023 [record in progress].*

## Aims

The aim of this study was to evaluate the clinical applicability of a Population pharmacokinetic (PPK) model for achieving Tac Co (therapeutic trough Tac concentration) versus the manufacturer’s labelling dosage.

## Interventions

Participants were randomised to either the PPK group or the control group with patients receiving Tac adjustment according to the manufacturer’s labeling.

## Participants

96 adult renal transplant recipients.

## Outcomes

The primary outcome was the percentage of patients reaching the Tac Co target (6 and 10 ng/mL) after the first steady state. The secondary outcomes were the timing needed to reach the therapeutic target, the number of dose modifications needed to reach the target, and the clinical outcome.

## Follow-Up

90 days posttransplantation.

## CET Conclusion

This single-centre randomised study compared initial tacrolimus dosing by body weight (control), or by Bayesian prediction (study), following renal transplantation. Patients in the study group had their tacrolimus dosing guided by a Bayesian model incorporating age, haematocrit and CYP3A genotype. The authors demonstrate that a significantly higher proportion of patients in the study arm achieved therapeutic target, with lower interpatient variability, shorter time to target trough concentrations and fewer dose modifications. Whilst no differences in clinical outcomes were seen, there was a trend towards lower incidence and shorter duration of DGF in the study group. These results are very promising and appear to demonstrate the benefit of personalised dosing using the Bayesian model. The population in this study are from a single centre, and predominantly male and Caucasian. Future studies should confirm these findings in populations with a greater mix of ethnicity, and confirm potential clinical benefit in a larger sample.

## Jadad Score

2.

## Data Analysis

Per protocol analysis.

## Allocation Concealment

No.

## Trial Registration

EudraCT—2016-000340-34

## Funding Source

Non-industry funded.

RANDOMISED CONTROLLED TRIAL 2 Immune monitoring-guided vs. fixed duration of antiviral prophylaxis against cytomegalovirus in solid-organ transplant recipients. A Multicenter, Randomized Clinical Trial.

*by Manuel, O., et al. Clinical Infectious Diseases 2023 [record in progress].*

## Aims

The aim of this study was to compare the effect of an immune monitoring–guided approach versus the current standard for tailoring the duration of antiviral prophylaxis to measure cytomegalovirus (CMV)-specific immunity in solid-organ transplant recipients.

## Interventions

Participants were randomised to receive a duration of antiviral prophylaxis according to immune–guided monitoring or a fixed duration (control).

## Participants

193 kidney and liver transplant recipients CMV-seronegative with seropositive donors or CMV-seropositive receiving antithymocyte globulins.

## Outcomes

The two primary endpoints were proportion of patients with clinically significant CMV infection and reduction in days of prophylaxis. The secondary endpoints were the incidence of all CMV events including untreated CMV replication, high-level CMV-DNAemia, patient survival, graft survival and incidence of acute rejection.

## Follow-Up

1 year.

## CET Conclusion

This multicentre trial enrolled kidney and liver transplant recipients receiving organs from CMV-positive donors, and randomised them to either fixed-duration prophylaxis, or guided by immune monitoring. In the study group, CMV ELISpot was used to monitor, and prophylaxis stopped if positive (indicating immune reactivity). The study failed to confirm non-inferiority of the immune monitoring strategy, although the overall rates of CMV infection were similar, with earlier CMV infection seen in the study group. However, duration of prophylaxis was shorter in the study arm. The failure to demonstrate non-inferiority is due to a lack of statistical power – in reality, the infection rates were very similar between groups. The study also fails to stratify randomisation by recipient serostatus, leading to an imbalance between the two arms of the study. This is important, as the risk of CMV infection is likely different between the two subgroups. Despite these limitations, it does appear that immune monitoring-guided prophylaxis is a reasonable strategy, resulting in a shorter duration of prophylaxis and a relatively low risk of clinically relevant CMV disease.

## Jadad Score

3.

## Data Analysis

Per protocol analysis.

## Allocation Concealment

Yes.

## Trial Registration

ClinicalTrials.gov—NCT02538172.

## Funding Source

Industry & non-industry funded.

## Clinical Impact Summary

This report is from a very interesting study in both liver and kidney transplantation, that could be practice changing. Monitoring for an immune response to CMV was used as a comparator to standard-duration CMV prophylaxis with valganciclovir. In the intervention arm of the study, prophylaxis was stopped if the immune monitoring showed a significant response (CMV ELISpot). The primary outcome was clinically significant CMV infection, which may be represented by symptomatic disease or asymptomatic viraemia that required treatment.

The study was designed on a non-inferiority basis and was statistically powered as such. Approximately 31% of patients had clinically significant CMV infection, which was higher than expected. This meant that the immune monitoring approach was not shown to be statistically non-inferior, despite similar event-rates in the study and control arms. The duration of antiviral prophylaxis was however, significantly shorter with immune monitoring, by about 26 days on average. The safety of the immune monitoring approach was consistent, whether or not the recipient was CMV positive or negative. The incidence of CMV disease was very low for both groups (0 versus 2 events). As the risk of any CMV infection was higher than expected in both arms, the 95% CI for the risk difference was wide and therefore a significant inferiority could not be ruled out.

Despite the limitations of the study, it seems that the immune monitoring strategy is safe and can result in a much earlier opportunity to stop CMV prophylaxis. A cost-benefit analysis would have been interesting to see but is not formally provided in this paper.

